# The efficacy of *Lactobacillus reuteri* DSM 17938 in infants and children: a review of the current evidence

**DOI:** 10.1007/s00431-014-2328-0

**Published:** 2014-05-13

**Authors:** Magdalena Urbańska, Hania Szajewska

**Affiliations:** Department of Paediatrics, The Medical University of Warsaw, 01184 Warsaw, Działdowska 1, Poland

**Keywords:** Probiotics, Randomized controlled trial, Systematic review

## Abstract

We aimed to systematically evaluate evidence on the effectiveness of *Lactobacillus reuteri* DSM 17938 (*L. reuteri*) for treating and preventing diseases in infants and children. MEDLINE and the Cochrane Library were searched in December 2013, with no language restrictions, for relevant randomized controlled trials (RCTs) and meta-analyses. The search was updated in April 2014. One systematic review and 14 RCTs met the inclusion criteria. The use of *L. reuteri* may be considered in the management of acute gastroenteritis as an adjunct to rehydration. There is some evidence that *L. reuteri* is effective in reducing the incidence of diarrhea in children attending day care centers. There is no evidence of effectiveness of *L. reuteri* in preventing nosocomial diarrhea in children. The administration of *L. reuteri* is likely to reduce crying time in infants with infantile colic in exclusively or predominantly exclusively breast-fed infants, but not in formula-fed infants. More studies are needed. Preliminary data suggest that *L. reuteri* may be effective in the prevention of some functional gastrointestinal disorders, such as colic and regurgitation. This innovative approach needs further evaluation by an independent research team. Preliminary evidence provides a rationale for further assessing the efficacy of *L. reuteri* for treating functional constipation or functional abdominal pain. However, it is too soon to recommend the routine use of *L. reuteri* for these conditions. There are no safety concerns with regard to the use of *L. reuteri* in nonimmunocompromised subjects. There are also data to support the safety of using *L. reuteri* in preterm infants. *Conclusion*: Our results precisely define current evidence on the effects of the administration of *L. reuteri* DSM 17938 to the pediatric population.

## Introduction

Probiotics, defined as live microorganisms, which, when administered in adequate amounts, confer a health benefit to the host [8] are usually discussed jointly. However, all probiotics are not created equal. Each strain has to be evaluated separately for its mechanisms of action, efficacy, and safety.

*Lactobacillus reuteri* DSM 17938 is the daughter strain of *L. reuteri* ATCC 55730. The latter was originally isolated from the breast milk of a Peruvian mother, and it may be present in normal humans on the mucosa of the gastric corpus and antrum, duodenum, and ileum [20, 30]. *L. reuteri* ATCC 55730 was found to carry potentially transferable resistance traits for tetracycline and lincomycin. Hence, it was replaced by *L. reuteri* DSM 17938, a strain without unwanted plasmid-borne resistance [23]. It remains a matter of debate whether or not *L. reuteri* DSM 17938, the strain with antibiotic resistance plasmids removed, and the original *L. reuteri* ATCC 55730 strain can be regarded as equal. If so, the data on *L. reuteri* ATCC 55730 can be extrapolated to *L. reuteri* DSM 17938. In principle, these two strains are not identical. However, there are studies, albeit limited, suggesting the bioequivalence of both strains. One in vitro study showed similarities with regard to the chromosomal genes, colony and cell morphology, fermentation pattern, mucin binding, and reuterin production [30]. Another study documented no differences between the strains in the characteristics of temporary colonization [6]. On the other hand, at least one study indicated that even the manufacturing process might influence the properties of probiotic bacteria. In that study, the Finnish group demonstrated that the differences in the in vitro properties of *Lactobacillus rhamnosus* GG isolates, in particular pathogen exclusion by inhibition and competition, depending on the product source (matrix) and production processes and conditions [11]. Whether or not these manufacturing differences translate into differences in vivo, as well as clinical outcomes, is a matter of discussion. The same uncertainty may apply to the impact of the removal of the plasmids.

Mechanisms of action of *L. reuteri* DSM 17938 and/or *L. reuteri* ATCC 55730 have been evaluated in a number of in vitro and animal studies. One of the best-documented mechanisms is their antimicrobial activity. *L. reuteri* strains produce reuterin, a broad-spectrum antibacterial substance [2, 29], which is capable of inhibiting the growth of a wide spectrum of microorganisms such as Gram-positive or negative bacteria, yeast, fungi, or parasites [3]. *L. reuteri* strains may also regulate immune responses. One such example is the modulation of TNF-α production from bacterial lipopolysaccharide (LPS)-activated monocytoid cells in a strain-dependent manner [15, 16]. With regard to *L. reuteri* strain DSM 17938, it was demonstrated that it improved LPS-induced intestinal morphological damage, including villus length and density [29]. Moreover, it has been demonstrated that *L. reuteri* DSM 17938 significantly reduced intestinal mucosal levels of KC/GRO (~IL-8) when newborn rats were fed cow milk formula plus *Escherichia coli* LPS [17]. Moreover, it has been demonstrated that *L. reuteri* DSM 17938 reduced intestinal inflammation in an experimental model of necrotizing enterocolitis via inhibiting a Toll-like receptor-4 signaling pathway that leads to cytokine expression [18]. Finally, some studies have suggested anti-inflammatory properties of *L. reuteri* DSM 17938. In vivo studies showed that this probiotic significantly reduced intestinal mucosal levels of proinflammatory cytokines (interleukin 8, interleukin-1β, interferon-γ, tumor necrosis factor alpha) in newborn rats with LPS-induced small intestinal and ileum inflammation [17]. Taken together, these data show that *L. reuteri* strains, like other probiotics, act through diverse mechanisms. These data also indicate a potential beneficial effect of *L. reuteri* strains in treating and preventing diseases in humans.

The aim of this review was to systematically review and update evidence on the efficacy and safety of using *L. reuteri* DSM 17938, regardless of the indication, in infants and children. The choice of the probiotic strain was determined by the facts that it is widely available and it is commonly used in the pediatric population.

## Methods

In December 2013, we searched MEDLINE and the Cochrane Library for relevant systematic reviews/meta-analyses of randomized controlled trials (RCTs) and subsequently published RCTs involving children aged 0 to 18 years. The search was updated in April 2014. The trials had to compare the use of *L. reuteri* DSM 17938 with placebo or no intervention. No language restriction was imposed. Terms used as a search strategy were as follows: *L. reuteri*, DSM 17938, *lactobacillus protectis*, child*, infant*, toddler*, adolescent*, and newborn*. Furthermore, the ClinicalTrials.gov website (http://clinicaltrials.gov/) and EU Clinical Trials Register website (https://www.clinicaltrialsregister.eu/) were searched for trials that were registered but not yet published. All clinical outcomes reported by the investigators if relevant to the current review were considered. One reviewer (MU) searched all databases and undertook data extraction. The risk of bias in the included studies was assessed using the Cochrane Collaboration’s tool for assessing risk of bias. The following criteria were used: type of randomization method (to assess the risk of selection bias), allocation concealment (selection bias), blinding of participants and personnel (performance bias), blinding of outcome assessment (detection bias), and presence of intention-to-treat analysis (attrition bias). A low risk of bias was indicated by an answer of “yes,” and a high risk of bias, by an answer of “no.”

## Results

The literature search yielded one systematic review and 14 RCTs. Table 1 summarizes the characteristics of the included trials. All included trials were double blinded, except one single-blinded trial. All were published in English. The ages of the children enrolled in the trials ranged from birth to 16 years. The daily dose of *L. reuteri* DSM 17938 ranged from 1 × 10^8^ to 4 × 10^8^ colony-forming units (CFU). With one exception, all RCTs were placebo controlled; in the remaining one, there was no additional intervention in the control group. The studies were undertaken in geographical Europe, the USA, Australia, and Mexico. Most of the included RCTs had a low risk of bias (see Table 2 for the methodological quality of the included trials). A list of excluded trials, mainly on *L. reuteri* ATCC 55730, is available upon request. These trials were excluded because of questionable bioequivalence with *L. reuteri* DSM 17938. Moreover, 17 trials that were registered, but not published, were identified. These included therapeutic or preventive studies on infantile colic (four RCTs), acute gastroenteritis (two RCTs), nosocomial diarrhea (two RCTs), asthma (one RCT), anorexia (one RCT), gastric motility in preterm newborns (one RCT), functional abdominal pain (two RCTs), chronic constipation (one RCT), functional constipation (one RCT), antibiotic-associated diarrhea (one RCT), and prevention of gastrointestinal and respiratory tract diseases (one RCT).

**Table 1:** Characteristics of included trials

Study ID (country)	Exp/cont	*L. reuteri* DSM 17938 (total daily dose, CFU)(duration of intervention)	Comparison	Inclusion criteria	Primary outcome(s)	Results	Funding
Management of acute gastroenteritis							
Francavilla et al. (Italy) [9]	37/37	4 × 10^8^ (for 7 days)	Placebo (mixture of sunflower oil and medium-chain triglyceride oil)	Age 6–36 months, clinical signs mild to moderate dehydration, not requiring parenteral rehydration	Duration of diarrhea, rate of unresolved diarrhea after 3 days of treatment	Duration of diarrhea: 2.1 ± 1.7 vs. 3.3 ± 2.1 days, *P* < 0.03	Not described
Dinleyici et al. (Turkey; Belgium) [5]	64/63	1 × 10^8^ (not described)	No intervention	Age 3–60 months, mild to moderate dehydration, hospitalization	Duration of diarrhea	MD −33.1 h, 95 % CI −42.6 to −23.6	Not described
Prevention of diarrhea							
Gutierrez-Castrellon et al. (Mexico) [12]	168/168	1 × 10^8^ (3 months)	Placebo (mixture of medium-chain triglyceride and sunflower oil)	Age 6–36 months, born at term, attending day care centers, without chronic disease, failure to thrive, allergy. or atopic disease	Number of days with diarrhea per child	0.32 vs. 0.96 days (*P* = 0.03)	BioGaia Sweden
Prevention of nosocomial diarrhea							
Wanke et al. (Poland) [31]	54/52	1 × 10^8^ (duration of hospitalization)	Placebo (not described)	Age 1–48 months, hospitalization for reasons other than diarrhea	Incidence of nosocomial diarrhea	RR 1.06, 95 % CI 0.7 to 1.5	Medical University of Warsaw
Management of infantile colic							
Savino et al. (Italy) [24]	25/25	1 × 10^8^ (21 days)	Placebo (not described)	Age 2–26 weeks, born at term, appropriate for gestational age, birth weight 2,500–4,000 g, exclusively breast-fed	Reduction of average crying time to <3 h/day on day 21	35 min/day (interquartile range 85) vs. 90 min/day (148), *P* = 0.022	BioGaia, Sweden
Szajewska et al. (Poland) [26]	42/40	1 × 10^8^ (21 days)	Placebo (not described)	Age <5 months, exclusively or predominantly (>50 %) breast-fed	Treatment success (percentage of children achieving a reduction in the daily average crying time ≥50 %), duration of crying (min/day)	Treatment success: day 7 (*P* = 0.026); day 14 (RR 4.3, 95 % CI 2.3 to 8.7); day 21 (RR 2.7, 95 % CI 1.85 to 4.1); day 28 (RR 1.6, 95 % CI 1.3 to 2.1)	Medical University of Warsaw
Sung et al. (Australia) [25]	85/82	1 × 10^8^ (1 month)	Placebo (maltodextrin)	Age <13 weeks, birth weight ≥2,500 g, no failure to thrive, no major medical problems, no cow’s milk protein allergy	Daily cry/fuss time (min/day) at 1 month	49 min/day higher in probiotic group in comparison to placebo group (95 % CI 8 to 90, *P* = 0.02)	Georgina Menzies Maconachie Charitable Trust, BioGaia Sweden, Calpro AS
Functional constipation							
Coccorullo et al. (Italy) [4]	22/22	1 × 10^8^ (8 weeks)	Placebo (not described)	Age >6 months, formula-fed, without organic disease, chronic disease, addition of pre- and probiotics to formula, laxatives and antibiotics intake	Bowel movements per week frequency, stool consistency, presence of inconsolable crying episodes	Bowel movements: week 2 (*P* = 0.042), week 4 (*P* = 0.008), week 8 (*P* = 0.027)	Noos, Italy
Functional abdominal pain							
Romano et al. (Italy) [22]	32/28	2 × 10^8^ (28 days)	Placebo (mixture of sunflower oil and a medium-chain triglyceride oil)	Age 6–16 years, FAP symptoms 4 weeks before potential study inclusion, without organic disease, chronic disease, growth failure	Reduction of the intensity of FAP	Week 4: 2.0 vs. 1.4, *P* < 0.001; week 8: 2.0 vs. 1.2, *P* > 0.05	BioGaia, Sweden
Regurgitation in infants with gastroesophageal reflux							
Indrio et al. (Italy) [14]	22/20	1 × 10^8^ (28 days)	Placebo (mixture of pharma-grade sunflower oil and a medium-chain triglyceride oil)	Age <4 months, formula-fed, normal growth and development, no underlying predisposing factors or conditions	Regurgitation frequency, median number of regurgitation episodes per day	1.0 (1.0 to 2.0) vs. 4.0 (3.0 to 5.0), *P* < 0.001	BioGaia, Sweden
Prevention of colic and regurgitation							
Indrio et al. (Italy) [13]	276/278	1 × 10^8^ (90 days)	Placebo (mixture of pharmaceutical-grade sunflower and medium-chain triglyceride oils)	Age <1 week on entry into the study, gestational age >37 and <41 weeks, birth weight adequate for gestational age, Apgar score >8 at 10 min, no congenital disorders and/or clinical or physical alterations at clinical examination	Presence of inconsolable crying episodes, duration of crying time	Day 30: 45 vs. 96 min/day, *P* < 0.01	BioGaia, Sweden
						Day 90: 38 vs. 71 min/day, *P* < 0.01	
					Regurgitation frequency, number of regurgitation episodes per day	Day 90: 2.9 vs. 4.6, *P* < 0.01	
					Bowel movements frequency, number of evacuation per day	Day 30: 4.01 vs. 2.8, *P* < 0.01	
						Day 90: 4.2 vs. 3.6, *P* < 0.01	
Garofoli et al. (Italy) [10]	20/20	1 × 10^8^ (28 days)	Placebo (not described)	Infants <3 days, full-term, breast-fed, no antibiotic or probiotic administration before inclusion	Daily regurgitation episodes	Week 2: (*P* = 0.05), week 4: (*P* = 0.02)	Noos, Italy
					Daily crying minutes	No statistically significant difference	
Preterm infants							
Rojas et al. (USA) [21]	372/378	1 × 10^8^ (duration of hospitalization)	Placebo (oil base)	Age <48 h, preterm infants, admission to the NICU, birth weight <2,000 g, hemodynamically stable	Death or nosocomial infection frequency	RR 0.87, 95 % CI 0.63 to 1.19	Not described
Oncel et al. (Turkey) [19]	200/200	1 × 10^8^ (duration of hospitalization)	Placebo (oil base)	Gestational age ≤32 weeks, birth weight ≤1,500 g, enteral feeding	Death beyond the 7th day of life and/or	RR 1.4, 95 % CI 0.76 to 2.59	Not described
					NEC stage ≥2 frequency	RR 1.26, 95 % CI 0.48 to 3.27	

*CFU* colony-forming units, *FAP* functional abdominal pain, *NEC* necrotizing enterocolitis, *NICU* neonatal intensive care unit

**Table 2:** Methodological assessment of included trials

Study ID	Adequacy of sequence generation	Allocation concealment	Blinding of participants and personnel	Blinding of outcome assessment	Incomplete outcome data
Francavilla et al. [9]	(+)	(+)	(+)	(+)	(+)
Dinleyici et al. [5]	(+)	(+)	(−)	(?)	(−)
Gutierrez-Castrellon et al. [12]	(+)	(+)	(+)	(?)	(+)
Wanke et al. [31]	(+)	(+)	(+)	(+)	(+)
Savino et al. [24]	(+)	(−)	(+)	(+)	(+)
Szajewska et al. [26]	(+)	(+)	(+)	(+)	(+)
Sung et al. [25]	(+)	(+)	(+)	(+)	(−)
Coccorullo et al. [4]	(+)	(?)	(+)	(?)	(?)
Romano et al. [22]	(+)	(+)	(+)	(+)	(?)
Indrio et al. [14]	(+)	(+)	(+)	(+)	(−)
Indrio et al. (2013) [13]	(+)	(+)	(+)	(+)	(+)
Garofoli et al. [10]	(+)	(?)	(+)	(?)	(?)
Rojas et al. [21]	(+)	(+)	(+)	(+)	(+)
Oncel et al. [19]	(+)	(+)	(+)	(+)	(+)

(+) indicates a low risk of bias, (−) indicates a high risk of bias, and (?) indicates unclear risk of bias

### Management of acute gastroenteritis

One recent systematic review [28] identified two RCTs [5, 9], one of which [5] was first published as the abstract only, but is now published as a full paper. In the first study [9], 74 children aged 6 to 36 months with acute diarrhea were randomized to receive *L. reuteri* DSM 17938 or placebo for 7 days. Compared with the placebo group, in the *L. reuteri* group, there was a significant reduction in the duration of diarrhea (3.3 ± 2.1 vs. 2.1 ± 1.7 days, respectively; *P* < 0.03), the risk of watery diarrhea on day 2 (81 vs. 55 %, respectively, *P* < 0.02) and day 3 (73 vs. 46 %, respectively, *P* < 0.03), and the risk of relapse of diarrhea (42 vs. 15 %, respectively; *P* < 0.03). There was not a significant difference in hospital stay between the groups.

The second RCT [5] involved 127 children aged 3−60 months with acute diarrhea who were randomly assigned to receive *L. reuteri* DSM 17938 or no intervention, both for 7 days. In comparison to the control group, the administration of *L. reuteri* DSM 17938 significantly reduced the duration of diarrhea (mean difference (MD) −33.1 h, 95 % confidence interval (CI) −42.6 to −23.6) and increased the risk of cure on day 3 (relative risk (RR) 6.2, 95 % CI 3.0 to 12.7). Moreover, the duration of hospitalization was reduced in the *L. reuteri* group compared with the control group (4.3 ± 1.3 vs. 5.5 ± 1.8 days, respectively; *P* < 0.001). Important study limitations include unclear adequacy of sequence generation, unclear allocation concealment, single blinding, and lack of intention-to-treat analysis. The pooled data from these two RCTs showed that compared with placebo or no treatment, *L. reuteri* DSM 17938 significantly reduced the duration of diarrhea (MD −32 h, 95 % CI −41 to −24) and increased the chance of cure on day 3 (RR 3.5, 95 % CI 1.2 to 10.8, random-effects model) [28]. The authors concluded that in hospitalized children, the use of *L. reuteri* DSM 17938 reduced the duration of diarrhea and more children were cured within 3 days. They also stated that data from outpatients and country-specific, cost-effectiveness analyses are needed. Moreover, given the limited data and the methodological limitations of the included trials, the evidence should be viewed with caution.

*In summary*, available data document the efficacy of *L. reuteri* DSM 17938. In line with recent guidelines developed by the Working Group on Probiotics of the European Society for Paediatric Gastroenterology, Hepatology and Nutrition, the use of *L. reuteri* DSM 17938 may be considered in the management of acute gastroenteritis as an adjunct to rehydration [27].

### Prevention of diarrhea

One RCT [12] assessed the effect of a daily administration of *L. reuteri* DSM 17938 for 3 months in preventing diarrhea in 336 otherwise healthy Mexican children aged 6 to 36 months attending day care centers. Compared with the placebo group, in the *L. reuteri* group there was a significant reduction in the number of episodes of diarrhea, episodes of diarrhea per child, mean duration of diarrhea episodes, and days with diarrhea per child both during the intervention and for the next 3-month follow-up period (the primary outcomes). Moreover, at both 3 and 6 months, there was a significant reduction in the number of respiratory tract infections (a secondary outcome). A cost-effectiveness analysis showed that intervention with *L. reuteri* DSM 17938 is cost saving for the community.

*In conclusion*, the findings from this trial suggest that administering *L. reuteri* DSM 17938 to children may be effective in reducing the incidence of diarrhea. These findings are of importance, as children attending day care centers are at greater risk for developing gastrointestinal and respiratory tract infections than children who stay at home. Earlier, Agustina et al. [1] showed in Indonesian children that the consumption of regular calcium milk (~440 mg/day) with *L. reuteri* DSM 17938 reduced the risk of diarrheal disease, particularly in malnourished children. Although a direct comparison of studies is difficult due the double-intervention in the study by Agustina et al., the findings from both trials are encouraging and suggest a means for preventing diarrheal diseases in children attending day care centers.

### Prevention of nosocomial diarrhea

One RCT [31] examined the effect of administering *L. reuteri* DSM 17938 for preventing nosocomial diarrhea in 106 children aged 1 to 48 months. Compared with placebo, the administration of *L. reuteri* did not significantly affect the risk of developing nosocomial diarrhea, defined as 3 or more loose or watery stools per 24 h occurring >72 h from the time of admission to the hospital (RR 1.06, 95 % CI 0.7 to 1.5) or rotavirus infection (RR 1.04, 95 % CI 0.6 to 1.6). There was also no difference between the probiotic and placebo groups for any of the other secondary outcomes (i.e., incidence of rotavirus infection, incidence of diarrhea, duration of diarrhea, incidence of recurrent diarrhea, incidence of chronic diarrhea, length of hospital stay in days, and frequency of need for rehydration).

*In conclusion*, there is no evidence of effectiveness of *L. reuteri* DSM 17938 in preventing nosocomial diarrhea in children. Further research is needed. One such RCT is currently underway (NCT01968408).

### Management of infantile colic

Three RCTs [24, 25, 26] evaluated the effect of *L. reuteri* DSM 17938 for the management of infantile colic. In the first RCT [24], researchers randomized 50 exclusively breast-fed infants with infantile colic according to the modified Wessel’s criteria to receive *L. reuteri* DSM 17938 or placebo for 21 days. Compared with the placebo group, in the probiotic group, the daily crying time was significantly reduced on day 21 [90 min/day (interquartile range 148) vs. 35 min/day (interquartile range 85), respectively; *P* = 0.022], and there was a significantly increased number of responders (defined as 50 % reduction in crying time from baseline) on day 7 (8 vs. 20, respectively; *P* = 0.006), day 14 (13 vs. 24, respectively; *P* = 0.007), and day 21 (15 vs. 24, respectively; *P* = 0.036).

The second RCT [26] involved 80 exclusively or predominantly (>50 %) breast-fed infants aged <5 months with infantile colic, also according to the modified Wessel’s criteria. These infants were randomly assigned to receive *L. reuteri* DSM 17938 or placebo for 21 days. Treatment success, defined as the percentage of children achieving a reduction in the daily average crying time ≥50 %, was significantly higher in the probiotic group compared with the placebo group at day 7 (*P* = 0.026), at day 14 (RR 4.3, 95 % CI 2.3 to 8.7), at day 21 (RR 2.7, 95 % CI 1.85 to 4.1), and at day 28 (RR 1.6, 95 % CI 1.3 to 2.1). In addition, throughout the study period, there was a significant reduction in the median crying time and in the parental perception of colic severity for parents of the infants in the probiotic group compared with the placebo group. Also, visual analog scale (VAS) scores showed improved parental/family quality of life throughout the study for parents and families of infants in the probiotic group compared with the placebo group.

One recent RCT [25] questioned earlier findings. In this trial, breast- or formula-fed infants (age <3 months) presenting with infantile colic were randomized to receive daily *L. reuteri* DSM 17938 or placebo. Of the 167 randomized infants, data from 127 (76 %) were analyzed. At 1 month, the mean daily crying or fussing time had fallen in both groups. However, compared with the placebo group, in the probiotic group, the daily crying time was significantly higher (mean difference 49 min/day, 95 % CI 8 to 90). This was mainly due to more fussing (MD 52 min/day, 95 % CI 19 to 84), as the crying time was similar in both groups (MD −2 min/day, 95 % CI −28 to 24). *L. reuteri* was not effective in improving infant sleep, maternal mental health, family or infant functioning, or quality of life.

**Fig. 1:**
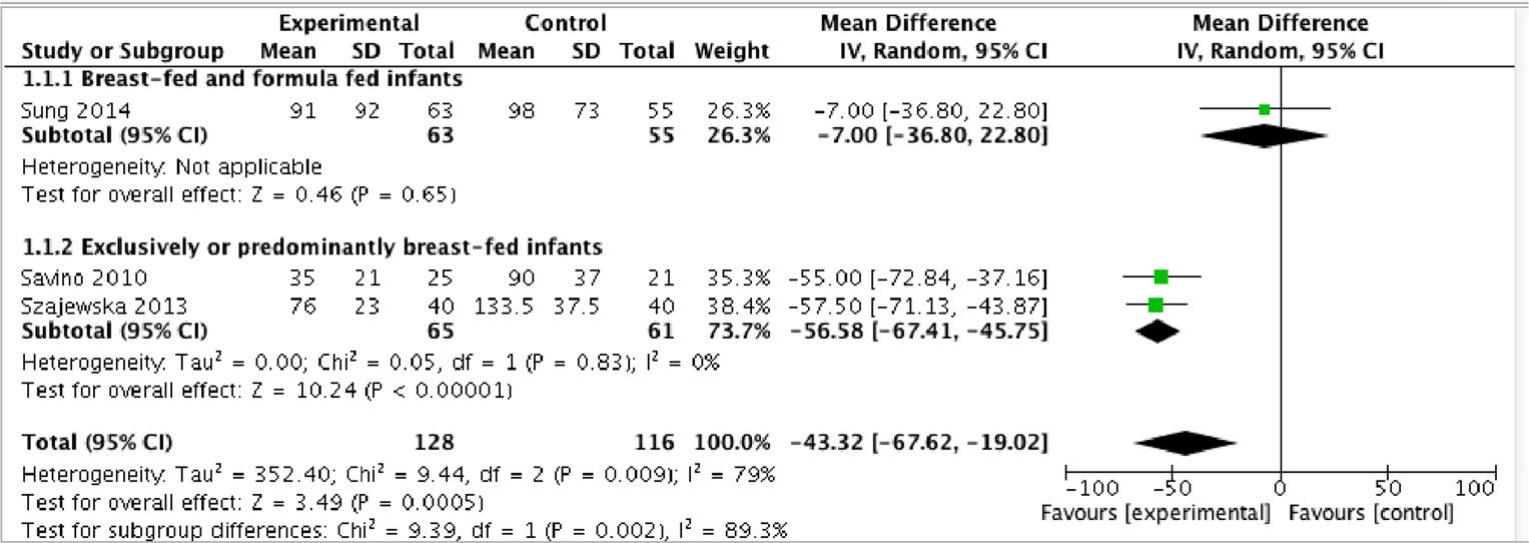
Infantile colic. *L. reuteri* DSM 17938 compared with placebo—effect on crying time on day 21

All three RCTs reported data on crying time on day 21. Here, we present the pooled results of three RCTs involving 244 infants. Compared with placebo, the administration of *L. reuteri* DSM 17938 reduced crying time on day 21 by approximately 43 min (MD −43 min/day, 95 % CI −68 to −19) (Fig. 1). This was mainly seen in exclusively or predominantly breast-fed infants (MD −57 min/day, 95 % CI −67 to −46).

*In conclusion*, the administration of *L. reuteri* DSM 17938 is likely to reduce crying times in infants with infantile colic, especially in exclusively or predominantly exclusively breast-fed infants. More studies, especially in formula-fed infants, are needed.

### Functional constipation

One RCT [4] involved 44 infants aged ≥6 months with functional constipation according to the Rome III criteria who were randomly assigned to receive *L. reuteri* DSM 17938 or placebo for 8 weeks. The primary outcomes were frequency of bowel movements per week, stool consistency, and presence of inconsolable crying episodes. Compared with the placebo group, infants in the *L. reuteri* group had a significantly higher frequency of bowel movements at week 2 (*P* = 0.042), week 4 (*P* = 0.008), and week 8 (*P* = 0.027). Throughout the study period, there was no statistically significant difference in stool consistency or inconsolable crying episodes between the study groups.

*In conclusion*, this limited data do not allow the routine use of *L. reuteri* DSM 17938 in the management of infants with constipation. Confirmatory studies are needed, particularly considering the limitations of the study (small sample size, unclear allocation concealment, unclear blinding of outcome assessment).

### Functional abdominal pain

One double-blind RCT [22] assessed the effect of *L. reuteri* DSM 17938 in the treatment of functional abdominal pain (FAP) according to the Rome III criteria. Sixty children were randomly assigned to receive either *L. reuteri* DSM 17938 or an identical placebo for 4 weeks followed by a 4-week follow-up period without supplementation. The primary outcome was the reduction of the intensity of FAP (Wong-Baker Faces scale). Compared with the placebo group, in the probiotic group, FAP intensity decreased significantly at weeks 4 and 8 (week 4: 1.4 vs. 2.0; *P* < 0.001, week 8: 1.2 vs. 2.0; *P* > 0.05). There was no difference in FAP symptoms frequency between the probiotic and the placebo groups.

*In conclusion*, limited evidence suggests that *L. reuteri* DSM 17938 may be useful for treating children with FAP. Further research is needed. One such RCT is currently underway (NCT01719107).

### Regurgitation in infants with gastroesophageal reflux

One double-blind RCT [14] showed that compared with the administration of placebo (*n* = 15), the administration of *L. reuteri* DSM 17938, for 30 days, to 19 formula-fed infants with regurgitation defined according to the Rome III criteria (≥2 regurgitations per day for ≥ weeks in otherwise healthy children) significantly reduced the median number of regurgitation episodes per day [4.0 (3.0 to 5.0) vs. 1.0 (1.0 to 2.0), respectively; *P* < 0.001]. Moreover, compared to the placebo group, in the probiotic group, the median fasting antral area was significantly reduced and the delta in the gastric emptying rate was significantly increased at the end of the intervention period.

*In conclusion*, limited available evidence suggests that *L. reuteri* DSM 17938 may help in decreasing regurgitation episodes and improving gastric motility in infants with gastroesophageal reflux, but more studies are needed.

### Prevention of colic and regurgitation

Two double-blind RCTs studied the prophylactic effect of *L. reuteri* DSM 17938 [13, 10].

One small RCT conducted in 40 breast-fed infants who received *L. reuteri* DSM 17938 or placebo for the first 28 days of life found similar durations of crying time, stool frequency, and stool consistency in both groups. However, compared to placebo, the administration of *L. reuteri* DSM 17938 significantly reduced the number of daily regurgitation episodes at the end of the treatment (*P* = 0.02) [10].

In the second, multicenter, large RCT [13], a total of 554 term-born, otherwise healthy breast-fed or formula-fed infants, aged less than 1 week, were randomly assigned to receive *L. reuteri* DSM 17938 or placebo for 90 days. Compared to placebo, the administration of *L. reuteri* DSM 17938 resulted in a significant reduction in crying time at 30 days (96 vs. 45 min/day, respectively; *P* < 0.01) and at 90 days (71 vs. 38 min/day, respectively, *P* < 0.01). Moreover, compared to placebo, the administration of *L. reuteri* DSM 17938 resulted in a significant reduction in the number of regurgitation episodes per day, but only on day 90 (4.6 vs. 2.9, respectively; *P* < 0.01), and a significant increase in the number of evacuations per day at day 30 (2.8 vs. 4.01, respectively; *P* < 0.01) and at day 90 (3.6 vs. 4.2, respectively; *P* < 0.01) [13].

*In conclusion*, for the first time, it was documented in an RCT that *L. reuteri* DSM 17938 was effective for preventing common functional disorders in infants, particularly infantile colic and regurgitation, in both breast-fed and formula-fed infants. Replication of these results is needed.

### Prevention of necrotizing enterocolitis

Two RCTs were identified that evaluated the effect of *L. reuteri* DSM 17938 for preventing necrotizing enterocolitis (NEC), nosocomial infections, and sepsis in preterm infants.

In the first RCT [21], the investigators examined the effects of administering *L. reuteri* DSM 17938 compared with placebo from the time of enrollment in the first 48 h of life until death or discharge to 750 preterm infants ≤2,000 g. Overall, there was no significant difference between the probiotic and placebo groups in the frequency of death or nosocomial infection (RR 0.87, 95 % CI 0.63 to 1.19), death (RR 0.8, 95 % CI 0.47 to 1.37), nosocomial infections (RR 0.88, 95 % CI 0.61 to 1.28), bloodstream infections (RR 1.44, 95 % CI 0.78 to 2.63), positive cultures (RR 0.86, 95 % CI 0.56 to 1.33), nosocomial pneumonia (RR 0.48, 95 % CI 0.22 to 1.05), nosocomial urinary tract infections (RR 2.37, 95 % CI 0.62 to 9.10), nosocomial meningitis (RR 1.02, 95 % CI 0.06 to 16.20), NEC (RR 0.6, 95 % CI 0.27 to 1.38), or episodes of feeding intolerance (RR 0.7, 95 % CI 0.4 to 1.09). However, in the subgroup of premature infants ≤1,500 g, there was a significantly reduced feeding intolerance (*P* = 0.04) and duration of hospitalization (*P* = 0.03).

In the second RCT [19], researchers randomized 424 preterm infants, with a gestational age of ≤32 weeks and a birth weight of ≤1,500 g, to receive *L. reuteri* DSM 17938 or placebo from the time of first feeding until discharge. In the whole study population, compared to placebo, the administration of *L. reuteri* DSM 17938 had no effect on the frequency of NEC stage ≥2 (RR 1.26, 95 % CI 0.48 to 3.27) or the frequency of death or NEC (RR 1.4, 95 % CI 0.76 to 2.59). However, in the probiotic group compared with the placebo group, there was a significantly reduced risk of proven sepsis (*P* = 0.041), full feeding day (*P* = 0.006), rate of feeding intolerance (*P* = 0.015), and duration of hospital stay (*P* = 0.022).

**Fig. 2:**
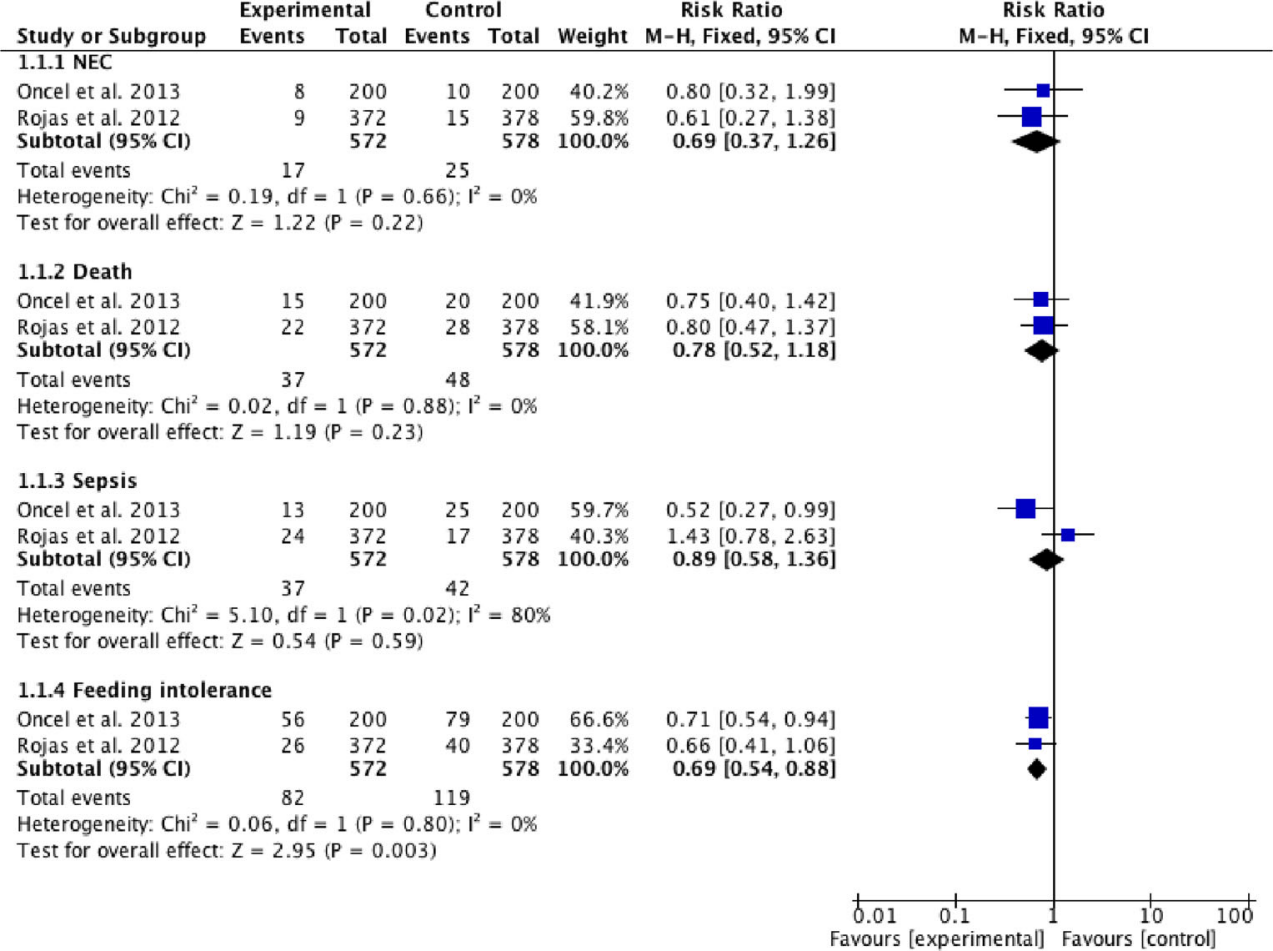
The effect of *L. reuteri* DSM 17938 compared with placebo on necrotizing enterocolitis (NEC), death, sepsis, and feeding intolerance episode frequency in preterm infants

Here, we present the pooled results of these two RCTs involving 1,150 preterm infants. Compared with placebo, the administration of *L. reuteri* DSM 17938 had no significant effect on the risk of sepsis, NEC, or death. However, there was a significant reduction in the risk of feeding intolerance (RR 0.69, 95 % CI 0.54 to 0.88) (Fig. 2).

*In conclusion*, the use of probiotics for preventing NEC is one of the most debatable indications for the use of probiotics. Certain probiotics prevent NEC; however, pooled data failed to demonstrate that *L. reuteri* DSM 17938 is one of them.

### Adverse events

*L. reuteri* DSM 17938 was well tolerated, and no adverse events associated with its administration were reported in any of the included trials. Two RCTs [14, 24] assessed growth parameters and found no difference between the probiotic and placebo groups. Moreover, in two RCTs carried out in preterm infants [19, 21], no probiotic grew from blood cultures.

*In conclusion*, the use of *L. reuteri* DSM 17938 in children with no additional risk factors for adverse events was safe and well tolerated. Of note, in 2011, the US Food and Drug Administration (FDA) gave *L. reuteri* DSM 17938, added to term infant formula, the Generally Regarded as Safe (GRAS) status [7].

## Discussion

The objective of this review was to summarize current evidence on the efficacy and safety of using *L. reuteri* DSM 17938 in the pediatric population. The use of *L. reuteri* DSM 17938 may be considered in the management of acute gastroenteritis as an adjunct to rehydration. There is some evidence that *L. reuteri* DSM 17938 is effective in reducing the incidence of diarrhea in children attending day care centers. There is no evidence of effectiveness of *L. reuteri* DSM 17938 in preventing nosocomial diarrhea in children. The administration of *L. reuteri* DSM 17938 is likely to reduce crying times in infants with infantile colic who are exclusively or predominantly exclusively breast-fed. More studies in formula-fed infants are needed. Preliminary data suggest that the administration of *L. reuteri* DSM 17938 may be effective in the prevention of some functional gastrointestinal disorders, such as colic and regurgitation. This innovative approach needs further evaluation by an independent research team. Preliminary evidence provides the rationale for further assessing the efficacy of *L. reuteri* DSM 17938 for treating functional constipation or FAP. However, it is too soon to recommend the routine use of *L. reuteri* DSM 17938 in the management of these conditions. There are no safety concerns with regard to the use of *L. reuteri* DSM 17938 in nonimmunocompromised subjects. There are also data to support the safety of administering *L. reuteri* DSM 17938 to preterm infants; however, the evidence in very low birth weight infants (<1,000 g) is very limited.

One important strength of our review is that it focused on one type of a clearly defined, single-organism, probiotic microorganism, specifically *L. reuteri* DSM 17938. Thus, our results precisely define the effects of the administration of *L. reuteri* DSM 17938 in the pediatric population. However, there are also several limitations to this review. One major issue with any systematic review is the possibility of publication and other reporting biases. However, with regard to *L. reuteri* DSM 17938, both positive and negative RCTs are being published which reduces, although does not eliminate, the risk of publication bias. Due to the fact that only single RCTs or a very small number of RCTs were available, formal assessment of the publication bias was not feasible. As stated earlier, for some conditions, only single trials were available, and confirmatory studies are, therefore, needed. Not only the number of trials but also the sample sizes in some trials were small. Another limitation is that the methodological quality of the included trials was variable.

In conclusion, well-conducted clinical studies using validated outcome measures are recommended to further identify populations that would benefit most from *L. reuteri* DSM 17938 administration. More studies are also needed if the efficacy of *L. reuteri* DSM 17938 was proven in a single trial only. Moreover, studies to evaluate the mechanisms of action of *L. reuteri* DSM 17938 are needed.

## Abbreviations

CI: Confidence interval
CFU: Colony-forming units
FAP: Functional abdominal pain
LPS: Lipopolysaccharide
MD: Mean difference
NEC: Necrotizing enterocolitis
RCT: Randomized controlled trial
RR: Relative risk
VAS: Visual analog scale
